# Exploring the Barriers to and Facilitators of Using Virtual Reality Relaxation for Patients With Psychiatric Problems: Qualitative Focus Group Study

**DOI:** 10.2196/65308

**Published:** 2025-06-11

**Authors:** Lisanne M Robbemond, JWH Mathijs Nijland, Manna Alma, Wim Veling, Catheleine MG van Driel

**Affiliations:** 1 Department of Psychiatry University Medical Center Groningen University of Groningen Groningen The Netherlands; 2 Department of Health Sciences University Medical Center Groningen University of Groningen Groningen The Netherlands; 3 VRelax bv Groningen The Netherlands

**Keywords:** virtual reality, relaxation, implementation, psychiatry, stress, qualitative research, barriers and facilitators, qualitative analysis, artificial intelligence, AI

## Abstract

**Background:**

Stress is a key transdiagnostic factor in the onset and recurrence of psychiatric disorders. Virtual reality (VR) in mental health care, particularly virtual natural environments, shows promising results in promoting relaxation, as evidenced by tools such as VRelax. While initial studies have demonstrated the efficacy of VRelax, further implementation in routine mental health care requires a systematic exploration of its use by patients. Understanding these perspectives can help tailor VR tools to meet the needs of users better and enhance their effectiveness in clinical settings.

**Objective:**

This study aims to identify patient-perceived barriers to and facilitators of using VR relaxation as a self-management relaxation tool to support its implementation in mental health care.

**Methods:**

Four focus groups were conducted with 19 participants with a wide range of psychiatric problems, including burnout, attention-deficit/hyperactivity disorder, anxiety disorder, depressive disorder, bipolar disorder, obsessive-compulsive disorder, and posttraumatic stress disorder. Participants were recruited via a network of people with lived experience, health care professionals, and social media. Semistructured interview guides with open-ended questions were used to investigate barriers and facilitators. People with psychiatric problems were instructed to use VRelax at home at least 3 times before the focus group discussions. Thematic analysis was conducted to identify barriers and facilitators.

**Results:**

The focus group discussions generated 7 themes with various subthemes. The sense of autonomy was identified as a facilitator, indicating users’ experience of feeling in control and independent, which allowed them to manage and operate VRelax on their own. On the other hand, participants indicated that for optimal long-term use, there should be a balance between autonomy and structured guidance and integration into therapy. Perceived usefulness, ease of use, and immersive factors were identified as both barriers and facilitators. Participants had positive initial experiences with VRelax but also reported that the effects of virtual natural environments might diminish with continued use. Usefulness might vary by the phase of psychiatric problems and the individual’s momentary emotional state. Participants saw the plug-and-play design of VRelax as helpful but also indicated the importance of easy navigation within the program, including the ability to quickly find specific natural environments. Three barriers were identified: shortcomings in user guidance; perceived problems in transitioning back to reality; and physical hindrances, such as the discomfort caused by the VR glasses.

**Conclusions:**

For optimal implementation of VR relaxation in mental health care, personalized VR experiences should be facilitated, such as offering a specific selection of virtual natural environments based on the momentary emotional state, while fostering user autonomy. Integration of VR tools into ongoing treatment is important, aligning with shared decision-making principles. In addition, reducing the steps required during the starting and the closing processes is crucial, alongside addressing challenges such as physical discomfort and inadequate instructions.

## Introduction

### Background

Psychiatric problems encompass various conditions affecting cognitive, emotional, and behavioral functioning that impact daily life and well-being [1]. Psychiatric problems can vary in severity and include psychiatric diagnoses, such as mood disorders, anxiety disorders, and psychotic disorders. In the Netherlands, almost half of people aged between 18 and 75 years have received more than 1 psychiatric diagnosis in their lifetime [2]. Mood disorders and anxiety disorders are the most prevalent disorders [2]. Psychiatric problems and stress have an intricate interplay. Stress responses, although often adaptive, can also increase vulnerability to disease when the response is recurrent or persistent over time [3]. Stress is a transdiagnostic factor associated with the onset, progression, and recurrence of various psychiatric disorders, such as anxiety and depression [4-6]. This study investigates the implementation of a virtual reality (VR) relaxation intervention that targets stress by exploring the needs of individuals who are experiencing psychiatric problems.

For many years, various types of relaxation exercises have been used for stress reduction, with moderate effects in patients with depression or anxiety [7]. More recently, mindfulness-based interventions have been introduced, aiming to reduce stress by incorporating various elements of mindfulness practices, psychoeducation, and relaxation techniques [8-11]. Mindfulness practices involve the direction of attention to mental representations, such as images and words, which appear to be beneficial for reducing symptoms of anxiety and depression [12,13]. Other stress management techniques, such as yoga and meditation, also seem promising in reducing stress [14,15]. Implementing these techniques can be challenging due to the high time investment required from health care professionals and the effort required from patients in the form of attention, concentration, imagination, and energy. This is particularly noteworthy considering that cognitive functioning in patients dealing with mental health problems is often impaired [16].

These limitations may be overcome by implementing more easy-to-use and immersive techniques to reduce stress in mental health care, such as VR, as it engages the user’s auditory and visual senses without much cognitive effort. VR refers to computer-generated environments that can be experienced through a head-mounted display or other specialized equipment. In a VR experience, individuals enter computer-generated simulations of real-life situations and are typically able to interact with and navigate through these simulated environments, which generate a strong sense of presence in the virtual environment [17,18]. These immersive experiences induce emotional, cognitive, and behavioral responses that can be used for therapeutic purposes. Creating numerous simulations opens the possibility of applying VR in various settings, including mental health care for stress reduction. Indeed, VR relaxation was found to be feasible, acceptable, immersive, realistic, easy to use, and usable in any location for the general population and adults with mental health conditions [19,20]. A recent study explored the feasibility of SafeVRwards, an evidence-based conflict-containment framework using virtual natural environments, showing potential benefits in enhancing relaxation and managing conflict in inpatient psychiatric wards [21].

Exposure to natural environments, both in VR and outdoors, to induce relaxation shows promising results [22-26]. The attention restoration theory suggests that exposure to nature can reduce stress, improve mood, and increase physical activity [27-29]. Moreover, the stress reduction theory posits that exposure to natural environments can impact emotional states by engaging the parasympathetic nervous system, leading to decreased stress levels and reduced autonomic arousal [30,31]. Research also suggests that nature sounds facilitate recovery in an individual after being exposed to a psychological stressor [32]. Therefore, a VR relaxation tool featuring natural environments was developed (VRelax). VRelax includes a collection of immersive 360° natural environment videos with slow gaming elements, designed to help individuals with mental or physical health problems relax and reduce their level of stress. VRelax is a self-management and plug-and-play tool that can be used autonomously in any location without the involvement of a health care professional. It has been shown to reduce perceived stress immediately in a range of populations, such as people with psychiatric disorders, intensive care nurses during the COVID-19 pandemic, and people with burnout [33-35]. Two weeks of VRelax use was effective in immediately improving perceived stress levels and both positive and negative affective states in patients with a psychiatric disorder [35]. A systematic review by Riches et al [20] identified 18 studies on VR relaxation in clinical populations, encompassing depression, bipolar disorder, and psychosis. The findings suggest that VR relaxation effectively promotes relaxation and reduces perceived stress in individuals with mental health problems.

Despite evidence supporting the effectiveness of VR relaxation in immediate stress reduction and its potential to reduce chronic stress and improve affective states, it is not widely implemented in everyday clinical practice. The current implementation of VR in health care settings indicates that, from the therapist’s perspective, barriers slightly outweigh facilitators across the technology, user experience, organizational context, and broader system [36]. In psychiatric care, clinical, organizational, and professional factors were identified by healthcare professionals [37]. The facilitators included the ease of use, the added value of VR, the positive attitude toward VR from patients and colleagues, the support for therapists, and the innovation-mindedness of the mental health care organization [34,36,37]. Barriers ranged from VR system limitations, limited time, insufficient technical support, and sustainability of VR over a longer period [34,36,37]. From the patient’s perspective, exposure to nature sounds and being able to personalize the VR experience seem to facilitate the relaxation process [32,38]. The barriers that patients experience range from a lack of personalization to the triggering effects of specific imagery, which can evoke negative memories or associations [34,38].

### Objectives

To aid the implementation of a self-management VR relaxation tool in mental health care, it is important to understand the barriers to and facilitators of using VR relaxation within this setting. Understanding these factors can inform the development of tailored implementation strategies and contribute to better mental health outcomes for patients. Therefore, the objective of this implementation study is to identify the barriers to and facilitators of using VRelax as perceived by patients with psychiatric problems.

## Methods

### Study Design

This implementation study used a qualitative design based on focus groups to optimize the VRelax application and its current clinical implementation as well as inform the design and conduct of a larger randomized controlled trial investigating the effectiveness of VRelax. The decision to use focus groups was motivated by their capacity to foster interaction among participants, thereby facilitating the emergence of important themes that might be missed in individual interviews with a more structured interview guide [39]. In addition, participants selected for the focus groups often shared certain characteristics, which allowed for the exploration of both shared and different views between participants [40]. The COREQ (Consolidated Criteria for Reporting Qualitative Research) checklist was used for the study design and reporting [41].

### Ethical Considerations

The Medical Ethics Review Board of the University Medical Center Groningen exempted this research from a full review and stated that this study did not fall under the Medical Research Involving Human Subjects Act (METc 2021/297). This study was conducted in accordance with the Declaration of Helsinki, particularly concerning scientific research, privacy, data management, and related ethical guidelines. Participants gave written informed consent before starting the focus group. All data were anonymized before analysis to ensure participant confidentiality. The participants received a financial reimbursement of €40 (US $45.35) for the time invested.

### Participants

A total of 4 focus groups were conducted with 19 participants between May 2021 and July 2021. Participants were selected if they were aged >18 years; had a psychiatric disorder according to the *Diagnostic and Statistical Manual of Mental Disorders, Fifth Edition*, which their health care provider diagnosed; received treatment in a general or specialized mental health care setting; reported that they were experiencing stress concerns; and expressed interest in using VRelax. Exclusion criteria were a history of epilepsy and brain injury with current concerns, as both conditions could potentially trigger seizures or other neurological events when using VR.

For the recruitment of participants, the researcher used a snowball sampling strategy [42] and recruited participants via the following routes: (1) the network of people with lived experience, (2) health care professionals in the north of the Netherlands, and (3) Facebook (Meta Platforms, Inc) and LinkedIn (Microsoft Corporation). Information about our study was distributed via email and social media More detailed information about the study was provided to all participants when they indicated an interest in participating. Written informed consent was obtained from all participants before participating in the study.

### Procedure

A total of 4 face-to-face focus group interviews were carried out with people with psychiatric problems. After providing consent to participate in this study, the participants received instructions and the Oculus Quest 2 (Meta Platforms) headset with VRelax installed by a member of the research team. The participants were asked to use VRelax at home at least 3 times. The focus groups were scheduled after participants had used VRelax at home for at least 1 week and no more than 3 weeks.

**Figure 1:**
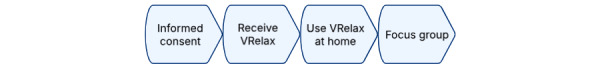
Flowchart of the procedure.

After receiving VRelax and using it at least 3 times at home, participants were invited to participate in a face-to-face focus group. Each focus group lasted approximately 120 minutes, including a small break. Focus group interviews were audio recorded with the consent of all participants. The moderator of the interviews was a member of the research team (LMR or JWHMN) who asked the questions from the interview guide and facilitated the interview. Both moderators, a psychologist (LMR, female moderator) and a psychiatrist (JWHMN, male moderator), were trained during practice focus groups on how to moderate a focus group interview. Neither had a previous relationship with any of the participants nor were they involved in the development or commercialization of VRelax. In addition, 1 of 2 people with lived experience was asked to attend the focus groups as coleaders and clarify participants’ responses, increase the sense of equality, reduce the potential barriers to sharing their opinions about VRelax in the group discussions, and provide a summary. The summary was presented during the focus group to check whether all relevant topics were discussed. Figure 1 shows a flowchart of the procedure.

### VRelax

**Figure 2:**
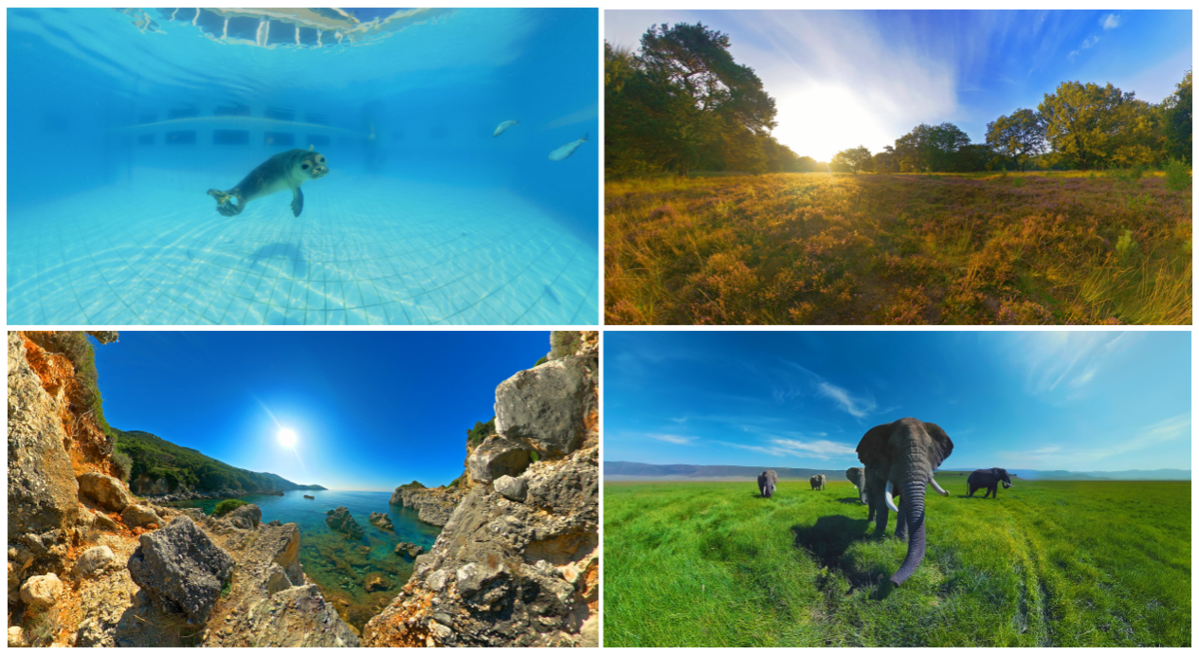
Impression of virtual relaxation natural environments from VRelax.

VRelax is a VR application designed to reduce stress and promote relaxation with an immersive nature experience for mental and physical health problems. VRelax (version 1.1.0), provided on Oculus Quest 2, offers a diverse range of immersive 360° audio-visual natural environment videos and images. Users can explore serene landscapes; engage in underwater experiences, such as scuba diving with dolphins; and experience different forest environments (Figure 2). The audio consists of both binaural sounds and nature sounds, for example, sounds of the waves or dolphins. Navigation is done by head movements that can activate hot spots visible in the VR environments. In certain natural environments within VRelax, users can engage with interactive slow-gaming elements. These include audio tracks guiding users through meditation and muscle relaxation exercises as well as the ability to interact with the environment, such as popping animated air bubbles by looking at them.

### Data Collection

The semistructured interview guide was developed before the first focus group interview and focused on gaining information about the preferences and experiences using VRelax (Multimedia Appendix 1). In addition, participants were asked in detail about the positive and negative experiences in each phase of using VRelax: (1) at the start, (2) during the use, and (3) after the use. The open-ended questions in the interview guide made it flexible and adaptive, allowing for the exploration of participants’ perspectives. The interview questions were piloted with 2 people with lived experience, who were not included in the study, to check for clarity and comprehensibility. The focus group interviews were informal and conducted on a first-name basis, and participants were encouraged to converse with one another and contribute as much as they felt comfortable.

After each focus group, a short recap was conducted with one of the moderators (LMR or JWHMN) and the patient with lived experience to summarize the focus group and formulate the key takeaways. In addition, after each focus group, a conformity meeting was held between the 2 moderators (LMR and JWHMN) to reflect on whether the focus group went according to the interview guide and discuss whether a topic needed to be added to the interview guide. After 4 focus groups, a consensus meeting took place (LMR, JWHMN, and CMGvD, the latter being a psychiatrist and female member of the research team) to evaluate the results. No new topics were discussed in the fourth focus group, which made the research team conclude that data saturation had been reached.

### Data Analysis

All focus groups were audio recorded and transcribed verbatim using AmberScript software (AmberScript Global BV). Afterward, all these transcripts were anonymized. All transcripts were verified to ensure accuracy (LMR). An inductive thematic analysis based on the study by Braun and Clarke [43] was conducted on the collected qualitative data, that is, the transcripts of the focus groups, using ATLAS.ti (version 23.2.3; Lumivero). The thematic analysis consisted of 6 steps: becoming familiar with the data, generating codes, generating themes, reviewing themes, defining and naming themes, and producing the report [43].

First, the transcripts of 2 focus groups were independently coded by 3 researchers (LMR, JWHMN, and CMGvD), and these codes were compared and organized into a coding tree. Next, LMR completed further coding and developed a set of meaningful data units. All transcripts were coded by LMR and reviewed at least 2 times during consensus meetings with JWHMN and CMGvD. The analytic approach was inductive, meaning the coding process began without an explicit preexisting structure or framework from which themes were drawn. During multiple meetings, the themes were reviewed by LMR, JWHMN, CMGvD, MA, and WV and agreed upon as the final themes. These final themes are reported in this paper. Illustrative quotations were translated from Dutch by LMR and reviewed by JWHMN and CMGvD. The translations that were agreed upon are presented to illustrate the themes and findings.

## Results

### Description of the Sample

In total, 19 participants participated in the 4 focus groups. Their demographic characteristics are provided in Table 1. The participants’ median age was 45 (IQR 34-53) years, and most were female (n=10, 53%). Most (n=7, 37%) patients had multiple *Diagnostic and Statistical Manual of Mental Disorders, Fifth Edition* diagnoses. Due to the COVID-19 pandemic restrictions, individuals experiencing symptoms resembling COVID-19 were not allowed to participate, resulting in smaller focus group sizes. The total sample size aligned with the recommended standards for meaningful analysis [43].

During the analysis, we found 7 themes. The following themes were identified as both barriers and facilitators: perceived usefulness, ease of use, and immersive factors. One theme was identified as a facilitator: enhancing autonomy. A total of 3 themes were identified as barriers: shortcoming (initial) guidance, insufficient transition back to reality, and physical hindrances. Within all but 1 theme, subthemes were identified. Each subtheme is substantiated with quotes. The following characteristics are provided successively: quote number, participant number, and patient representative number. The definitions of each theme were derived from the results of the thematic analysis (Table 2). All original Dutch quotes are provided in Multimedia Appendix 2.

**Table 1:** Characteristics of the focus group (FG) participants^a^ (N=19).

Characteristics			FG 1 (n=5)		FG 2 (n=4)		FG 3 (n=5)		FG 4 (n=5)
Female sex, n (%)			2 (40)		3 (75)		3 (60)		2 (40)
Age (y), mean (SD)			45 (21.1)		46.8 (17.5)		43.6 (14.6)		45.6 (15.5)
**Psychiatric diagnoses^b^, n (%)**									
	ADD^c^ and ADHD^d^	—^e^		—		1 (20)		2 (40)	
	Anxiety disorder	—		2 (50)		—		2 (40)	
	Bipolar disorder	1 (20)		1 (25)		—		1 (20)	
	Depressive disorder	3 (60)		1 (25)		—		3 (60)	
	OCD^f^	—		—		1 (20)		—	
	Psychotic disorder	1 (20)		1 (25)		1 (20)		—	
	PTSD^g^	1 (20)		—		2 (40)		1 (20)	
	Burnout^h^	—		—		1 (20)		—	

^a^Not including people with lived experience.

^b^Participants were able to self-report >1 *Diagnostic and Statistical Manual of Mental Disorders, Fifth Edition* diagnosis and burnout, meaning the total number of reported diagnoses might exceed the number of participants.

^c^ADD: attention deficit disorder.

^d^ADHD: attention-deficit/hyperactivity disorder.

^e^Not applicable.

^f^OCD: obsessive-compulsive disorder.

^g^PTSD: posttraumatic stress disorder.

^h^Not an official *Diagnostic and Statistical Manual of Mental Disorders, Fifth Edition* diagnosis.

**Table 2:** An overview of the themes, definitions, and subthemes.

Theme	Barrier or facilitator	Definition	Subtheme
Perceived usefulness	Barrier and facilitator	The perception that users have regarding the value, effectiveness, and benefits of using VRelax and the factors that influence this	Barrier and facilitator: adaptation over time Barrier and facilitator: dynamic influence of internal factors Barrier and facilitator: impact of emotional associations
Ease of use	Barrier and facilitator	The experienced ease with which users interact with the VRelax software	Barrier: effort-reward imbalance Barrier: making targeted choices Barrier: malfunctions disrupting use Barrier and facilitator: plug-and-play nature
Immersive factors	Barrier and facilitator	These factors hinder or aid the user’s ability to fully immerse themselves in the virtual environment, potentially enhancing or diminishing the effectiveness of the relaxation experience	Barrier and facilitator: impact of audio on immersion Barrier and facilitator: interactions with animals and people in VR^a^ Barrier: impairments in realism Barrier: unable to let go of one’s own thoughts
Enhancing autonomy	Facilitator	The users’ desire for independence and control over their interactions with VRelax	Facilitator: facilitating autonomy Facilitator: possibility to retreat Facilitator: proximity
Shortcoming (initial) guidance	Barrier	The lack of effective and sufficient direction or instruction provided by a therapist to users during the use of VRelax	Barrier: (initial) instructions missing Barrier: integration with therapy goals
Insufficient transition back to reality	Barrier	The absence of a structured and supportive transition period within the VRelax experience to the real world and daily life	—^b^
Physical hindrances	Barrier	Physical attributes of the headset and the surrounding space or physical limitations that influence the use and experience of VRelax	Barrier: limited by physical space Barrier: low comfort of the VR headset Barrier: physical limitations Barrier: side effects

^a^VR: virtual reality.

^b^Not applicable.

### Theme 1: Perceived Usefulness

#### Adaptation Over Time

Positive feelings were expressed before participants’ first use of VRelax, namely curiosity toward VR, the VRelax app itself, and its effect on participants’ stress levels. Participants’ feelings and thoughts about VRelax included descriptions such as “curious” (quote 1; P15; male participant aged 57 y); “hopeful” (quote 2; P16; male participant aged 32 y); and “excitement, but positive excitement” (quote 3; P17; female participant aged 29 y). These feelings seemed to create a reason to start using VRelax.

Over time, participants transitioned from initial, first-time use of VRelax to more repeated engagement. This shift mostly involved a transition from experiencing positive or facilitating feelings to encountering more barriers or negative feelings related to continued use. After using VRelax a couple of times, participants mentioned the following:

In the beginning, it takes some time searching. I am also curious about what there is out there.

Quote 4; P7; male participant aged 72 y

I got bored quite quickly.

Quote 5; P2; male participant aged 36 y

Lacking appropriate stimuli or varied forms of stimuli in the environment led to a sense of boredom or monotony:

Later on, I also got that indefinable feeling and a bit of emptiness. I have tried a lot of things and have not been able to find anything that really suits me at that moment.

Quote 6; P5; male participant aged 78 y

You will find out what you like and don’t like, what you need.

Quote 7; P1; female participant aged 49 y

These feelings and experiences could either encourage continued use of VRelax or act as barriers that make it difficult to persist with its use.

#### Dynamic Influence of Internal Factors

The phase of the disorder (eg, manic, psychotic, depressed, and anxious) impacted the perceived usefulness of VRelax. One participant mentioned as follows:

So if I were experiencing psychosis, I certainly would not use it because it might cause more panic. Considering the depression I’ve gone through, I believe it could be effective then, serving as a silver lining or source of relaxation.

Quote 8; P3; female participant aged 21 y

Another participant added the following:

So you have different stages: you have a path towards depression, you have depression, and then you have after a depression. And I know in which situation those glasses [VRelax] are optimal for me. Sometimes people know they’re going to get depressed, they can feel it coming, and they can stop it if they use the glasses.

Quote 9; P4; male participant aged 78 y

Furthermore, participants’ perceptions of the effects of using VRelax varied depending on their momentary affective state (eg, discomfort and interest). It seemed that their momentary affective states depended on varying circumstances and internal factors. The perceived usefulness of using VRelax could either be viewed as beneficial or not beneficial for the participants:

I also noticed that there were moments when I had used VRelax, and it did not feel good for me. But if I had a stressful day, then VRelax was very pleasant.

Quote 10; P6; female participant aged 32 y

It varies each time, there is no one way. Sometimes you need to be active, sometimes you need to lie down and take the time to reflect, and sometimes you need to go outside or find distraction. It varies for me.

Quote 11; P6 female participant aged 32 y

#### Impact of Emotional Associations

Participants mentioned that certain virtual natural environments evoked personal memories and associated feelings, either facilitating or hindering the relaxation induced by these environments. In some cases, previous experience with a natural environment depicted in VRelax positively influenced the impact that the virtual natural environment had, even causing relaxation:

There is one nature environment, which is a lake with a forest. I often go to Terschelling [Wadden Island, island group in the Netherlands] and there is an ice-skating ring with a lake. So, when I was in the environment, I was thinking, is this recorded there? I found this a very pretty experience to enjoy.

Quote 12; P7; male participant aged 72 y

Participants reported that the virtual natural videos in VRelax triggered negative personal memories, hindering relaxation:

Sometimes using VRelax made me tense up particularly, with those pictures where you looked at images of landscapes. Childhood memories from my difficult phase in life came up, and I found it very unpleasant that they came up that way because of those stupid VR glasses. I am very stable then and you start to wonder if you are stable.

Quote 13; P4; male participant aged 78 y

### Theme 2: Ease of Use

#### Effort-Reward Imbalance

Participants stated that the effort involved in getting started with VRelax shaped how they perceived the rewards of using VRelax, especially the intended calming effect. When asked how participants felt about the amount of effort they had to put in before getting started, one participant described feelings of “irritation, especially in the beginning because I had to figure out how it works” (quote 14; P18; female participant aged 65 y). The amount of effort and the associated feelings acted as a barrier to the perception that participants had of the benefits of using VRelax:

Activating the stars and the breathing exercises required effort, which prevented the relaxation

Quote 15; P18; female participant aged 65 y

#### Making Targeted Choices

The application menu was described as “unclear” (P6; female participant aged 32 y) and causing feelings of “agitation” (quote 16; P1; female participant aged 49 y) due to the lack of information about each natural environment. This confusion was especially problematic when participants, who were already stressed, were trying to find a specific natural environment.

A few participants discussed as follows:

There is so much happening, but you actually have no idea what you will encounter in those videos.

Quote 17; P1; female participant aged 49 y

And perhaps the idea behind it was: you go on an adventure, through the app, you start discovering. But very often I do not have much time, I have a family, I have a job, and I am always busy. What I lack in a day is free time, so I just want to make targeted choices.

Quote 18; P2; male participant aged 36 y

I had heard that there is also a breathing exercise included and I wanted to find it. I suggest putting it in the instruction manual because I searched my butt off. I was already restless and then you get even more restless.

Quote 19; P15; male participant aged 57 y

#### Malfunctions Disrupting Use

Some participants encountered malfunctions while using VRelax, specifically during the closing process, for example, when answering the questions, which disrupted their overall VRelax experience. The questions presented during both the starting and closing processes asked about participants’ level of relaxation and calmness. These malfunctions caused frustration and hindered participants from achieving a relaxed state:

I found it difficult to get out of the program [VRelax]. I got stuck on those two questions. Then I could switch something off, but I ended up with those two questions again.

Quote 20; P4; male participant aged 78 y

#### Plug-and-Play Nature

The plug-and-play nature was related to immediate use without manual setup. Participants shared both positive and negative experiences related to the plug-and-play nature (or lack thereof) of VRelax, namely difficulties during the starting and closing processes, an unclear menu, information gaps, and the multitude of options in the application menu.

There were instances where the plug-and-play nature posed challenges, such as the difficulties in the starting and closing processes. These steps required time and effort to become accustomed to:

Getting started takes a really long time. I had to put the thing [VR headset] on and then turn it on. Then I had to enter my code in a virtual living room. Sometimes this went right, and sometimes it went wrong. Then I still have to pick an app I want.

Quote 21; P4; male participant aged 78 y

Other information gaps included missing details of the current time and the location of favorite natural environments:

I would like to be able to choose between one of three minutes or one of ten minutes. Because now I wonder, how long does this take? Does it fit in a short break, or should I take a more extensive period to do this?

Quote 22; P2; male participant aged 36 y

Conversely, some participants highlighted the high usability and intuitiveness as contributing factors to the ease of use:

I find it very positive that everything is very straightforward. I think it is quickly learnable for digital novices.... Once you get the hang of it, which happens very quickly, it works very smoothly.... This also encourages using it.

Quote 23; P4; male participant aged 78 y

The discovery of what is being offered and how to get into VRelax is all clear and inviting. I also found the ease of use to be ideal.

Quote 24; P5; male participant aged 41 y

### Theme 3: Enhancing Autonomy

#### Need for Autonomy

Several participants expressed satisfaction with the level of control they had within VRelax, identifying it as an important facilitator. They appreciated the ability to select their preferred natural environment that suited their needs, especially for the participants whose needs were influenced by their changing moods or other disease characteristics. Participants believed that being instructed on which natural environment to choose would not be beneficial for reducing their stress levels and might even have a counterproductive effect:

Because I’m personally in favor of people having the space to choose for themselves where they want to go. So not that the practitioner says: well, you’re depressed now, so then I’ll just give the depression movies so to speak. But that we as users can decide that ourselves works best for me.

Quote 25; patient representative 1; female participant aged 54 y

#### Possibility to Retreat

Some participants believed that using VRelax allowed them to retreat from their daily activities. The freedom of having the possibility to leave a certain situation or after a stressful event made the participants feel like they could go to a place that was their own, providing a sense of control and ownership:

I thought: wow, I have my own little space.

Quote 26; P6; female participant aged 32 y

In addition, most participants mentioned that they preferred to use VRelax during a time when there was enough peace and quiet:

I have two small children, one is quite big already, but I mean during the day they are very busy and then you do not start easily. You can still hear everything around you.... I did it when the kids were in bed and before I went to bed. I could relax and then go to sleep relaxed.

Quote 27; P10; female participant aged 46 y

#### Proximity

Several participants stated that they experienced the proximity of the VRelax headset at home as a facilitating factor:

It is purely learning to experience what it is, and the device is also always next to me, so the moment I want to do it, I can use it.

Quote 28; P7; male participant aged 72 y

### Theme 4: Immersive Factors

#### Impact of Audio on Immersion

The audio in VRelax seemed to affect the immersion both positively and negatively. One participant mentioned that the “monotonous sound” (quote 29; P1; female participant aged 49 y) helped them feel more immersed. In contrast, other participants found the audio “irritating” (quote 30; P4; male participant aged 78 y) and reported that it did not contribute to their sense of immersion.

#### Interactions With Animals and People in VR

VRelax featured multiple natural environments with either animals or a person with singing bowls. Some participants mentioned that they found these interactions scary because they were too close, while others found these environments useful:

About those elephants, I know that you shouldn’t get too close to them. I’m not afraid of animals, it’s not that, but I found it too big. Too close. In that sense, I find the animals too large.

Quote 31; P7; male participant aged 72 y

The horses and being in contact with them had a positive effect on me.

Quote 32; P18; female participant aged 65 y

#### Impairments in Realism

Participants identified various barriers with VRelax as sources of annoyance and disruption to the overall immersive experience, including the lack of smells associated with nature and low video quality:

I miss the smell, and then I thought, “Actually, I have that [smell] every day.” Like today, I went to the park with my dog, and then I saw the grass and the flowers while walking and thought, “Yes, this makes me feel relaxed.”

Quote 33; P18; female participant aged 65 y

I was annoyed by the image quality, which prevented me from experiencing full immersion.

Quote 34; P19; male participant aged 45 y

Participants also mentioned that the perspectives of the nature videos in VRelax were too high, as these videos were recorded from a standing viewpoint while users were seated during use, making them feel they were unrealistic:

My point of view is set too high in VRelax, which makes it unpleasant. The unrealistic imagery also makes it less conducive to relaxation.

Quote 35; P6; female participant aged 32 y

#### Unable to Let Go of Own Thoughts

During the discussion, it became clear that for some participants, their stream of thoughts was a barrier to using VRelax. The immersive factors of VRelax were not enough to let go of their thoughts and be fully immersed. These participants were still experiencing their continuous stream of thought, which prevented them from experiencing relaxation:

My thoughts just continued, causing significant distraction....Standing in the meadow and having horses come up to you, I know from personal experience that this works very calming..... [VRelax] failed in eliciting that feeling.

Quote 36; P19; male participant aged 45 y

### Theme 5: Shortcoming (Initial) Guidance

#### Instructions Missing

Before using VRelax, participants received information on how to use VRelax from a member of the research team and a written manual. One participant described this conversation as “very clear” (quote 37; P5; male participant aged 45 y). However, participants mentioned that they still missed practical information about the VR headset, the VR controllers, the effects of VR, and how to get started:

So, I needed time to get used to it, as I’ve mentioned before. It is kind of important that people are forewarned about this because I was one of those who needed time to adapt.

Quote 38; P6; female participant aged 32 y

#### Integration With Therapy Goals

The lack of instructions before using VRelax and guidance during its use seemed to create confusion regarding how to use VRelax and understand its goal. This acted as a barrier to incorporating VRelax into the therapy goals. Participants wanted the VRelax sessions to be evaluated together with their health care professionals:

I expect that I can discuss it regularly with a therapist, that it has some kind of follow-up. We then have a sort of end goal, and we work towards that.

Quote 39; P7; male participant aged 72 y

### Theme 6: Insufficient Transition Back to Reality

A few participants found that the lack of a transition back to the real world after using VRelax was a barrier. Many steps had to be taken in the closing process of VRelax, and there was a lack of guidance within VRelax. The inadequate guidance within the VRelax app made the process of readjusting to their physical surroundings challenging. This could even work counterproductively on the relaxed feeling. Participants experienced lingering sensations from the virtual environments in real life:

Yes, there were times when I had to adjust to where I was and to the lighting. Sometimes it was very abrupt to be back in reality. I sometimes had the feeling that I hadn’t quite come back from the virtual world, and there was that indefinable feeling too. I really had to recover for five minutes. I noticed after a few days that... when I closed my eyes, I still had a 3D space around me..... This is something I did not look forward to when I put it on again: what will play in my head this time.

Quote 40; P5; male participant aged 41 y

### Theme 7: Physical Hindrances

#### Low-Comfort VR Headset

Some participants found the Oculus Quest 2 VR headset uncomfortable and difficult to adjust to, which led to feelings of stress and distraction, hindering the ability of VRelax to reduce stress:

The first few times I was very occupied with adjusting the glasses. The pressure I was experiencing on my face kept me distracted. Instead of helping me to relax, the glasses [VRelax] remained constantly on my mind..... It is a shame, it would be nice to forget the glasses and imagine yourself purely in the VR world.

Quote 41; P13; male participant aged 44 y

#### Physical Limitations

A few participants specifically mentioned that their physical limitations, such as eye abnormalities, uncontrollable hand movements due to old age, and neck concerns, negatively impacted their use of VR:

I also have a lazy eye and then sometimes I had trouble focusing and that is very difficult with those [VR] glasses and is sometimes annoying because then I feel like I’m squinting.

Quote 42; P17; female participant aged 29 y

#### Side Effects

Participants experienced side effects, such as headaches or dizziness, which served as barriers to using VR for extended periods.

It made me very nauseous and gave me a headache so that I couldn’t put it [VRelax] on for more than five or ten minutes.

Quote 43; P3; female participant aged 21 y

## Discussion

### Principal Findings

This study aimed to identify the barriers to and facilitators of using VR relaxation for patients with psychiatric disorders. A total of 7 themes emerged from 4 focus groups, with 19 participants. Three themes were identified as barriers and facilitators: perceived usefulness, ease of use, and immersive factors. One theme was identified as a facilitator: enhancing autonomy. The remaining 3 themes were identified as barriers: shortcoming (initial) guidance, insufficient transition back to reality, and physical hindrances.

The themes of perceived usefulness, ease of use, and immersive factors, which are all identified as barriers and facilitators, highlight those individual needs shaped by psychiatric problems and current affective state. When these factors function as barriers, even a single use of VRelax can negatively impact the user experience and hinder long-term implementation in mental health care. These themes align with previous research on the importance of usability and engagement in VR-based interventions [44]. VRelax offers a wide range of virtual natural environments, allowing users to find an environment that suits their current state. Finding a virtual natural environment that fits the user’s preferences could improve their positive mood and create higher satisfaction [45,46]. However, the search for an environment is not always successful (eg, high arousal in combination with a natural environment that is too quiet and calm). Passive virtual experiences, such as watching nature scenes without interacting with the environment, seem to result in lower levels of cognitive engagement and interest over time [34,47,48]. Personalization in VR; adaptations in which virtual natural environments are provided based on the current individual needs of the possible users, for example, by asking about the current mood; and presenting only a selection of virtual natural environments positively contributes to relaxation and engagement [38]. These results are consistent with previous research on the need for personalization in VR [38,49,50].

A need to easily find specific virtual natural environments is reflected in the themes of perceived usefulness and ease of use. These 2 themes align with the technology acceptance model, which posits that perceived usefulness and ease of use have a direct effect on attitudes toward using technology and, ultimately, influence the intention to use technology [51]. However, the role of ease of use on the intention to use a given technology may be nuanced [52]. When there is a mismatch between the needs of patients and the virtual natural environments, the perceived usefulness will decrease, negatively impacting the attitude and intention to use VRelax. Participants mentioned that they wanted to easily select a suitable natural environment that suits their current internal state (eg, affective state and phase of disorder). Therefore, a simple user interface, in which the characteristics of the natural environments are visible and the favorite environments are easily accessible, could be beneficial.

The sense of autonomy reveals the need for users to have a sense of control and independence, such as the ability to manage and operate VRelax on their terms without relying on external assistance. In mental health care, the waitlists and people requiring mental health care are on the rise, which results in a higher demand for self-management tools. VRelax is intended to be used as a self-management tool alongside standard care, which seems to be an approach that is beneficial for people with psychiatric problems [53]. The sense of autonomy plays a role in the motivation to continue using technology [54]. Thus, the sense of autonomy provides patients with empowerment and better functioning in life and is beneficial for the implementation of a self-management tool.

Patients need both autonomy and structured guidance for the effective use of VRelax. Previous research has not explicitly examined the interplay between these 2 needs or the significance of maintaining the balance between them. Imbalances between these needs led to participant frustration, hindering long-term use. In addition, personalizing treatment to the patient’s needs and treatment goals as well as fostering control and self-efficacy were already reported as facilitators in the implementation [36]. To address these challenges, adaptive VR systems, using machine learning algorithms and generative artificial intelligence, can dynamically adjust the level of guidance and autonomy based on real-time patient responses and progress. This flexibility balances providing necessary structure and promoting patient agency, potentially enhancing treatment engagement and outcomes. Moreover, the ability to tailor VR experiences to individual preferences may increase the ecological validity of the interventions, making them more relevant and applicable to patients’ daily lives.

The results of this study also emphasize the importance of considering the therapist’s role in implementing VR relaxation into mental health care. Given the unfamiliarity with VR technology, it is crucial to ensure that both clients and clinicians receive appropriate information and ongoing support to use the technology effectively. Implementation packages should be developed for clinical settings, which include onboarding guides with practical information about the app and recommendations for integrating VRelax into work processes and treatment protocols. Furthermore, guidelines for safe and effective use would be helpful, based on clinical studies and professional experiences with VRelax. Finally, training materials for clinicians would promote implementation, such as online videos or workshops. Providing information on the therapeutic aim, rationale, safety of the VR technology, and its intended users might be beneficial for integrating the technology into standard care [34,55]. Moreover, taking into account whether the VR technology is suitable for the treatment aim and phase of treatment could be advantageous for the successful use of the VR technology.

The theme of physical hindrances, particularly discomfort with the VR headset, was identified as a significant barrier, as it caused frustration and directly impacted implementation and long-term use. Reducing these hindrances could enhance the usability and adoption of VRelax. Some participants reported side effects consistent with cybersickness, denoting bodily discomfort associated with exposure to VR content [56-58]. Cybersickness might be related to a low sense of presence in the VR environments [58]. Despite advancements in newer head-mounted displays showing fewer cybersickness problems, cybersickness is still being reported [56]. In addition, the hardware has been identified as a barrier. The weight of the Oculus Quest 2 can cause discomfort during the use of VRelax. Moving one’s head around and looking through lenses to see the whole virtual environment may prove difficult, especially when experiencing physical limitations. This concern aligns with findings by Brown [59], who used thematic analysis to explore the challenges and opportunities of VR use among older people. With future developments in VR headsets, the potential discomfort should be taken into account.

### Limitations

This is one of the first qualitative studies exploring the experiences of using VR for relaxation in patients with mental health problems. The purpose of the focus groups was to explore the barriers and facilitators patients experienced in using VRelax. The consistency of themes across the focus groups suggests that our sample size was adequate and data saturation was reached. However, several limitations must be considered when interpreting the results. This study relies solely on focus group discussions, without triangulating findings with other data sources, such as individual interviews, observational data, or application use metrics. There may be a selection bias stemming from participants’ specific interest in technology and VR and willingness to participate in focus groups, potentially limiting the generalizability of our findings to all patients with mental health care problems. We did not assess participants’ previous VR experience, which may have influenced perceived usability challenges and barriers. However, no one mentioned previous experience with VRelax or other VR technologies during focus groups. Furthermore, participants were not asked to rank the identified barriers and facilitators, which means that the relative importance of each was not investigated.

### Future Research

One of the main findings of this study indicates that a sense of autonomy is a facilitating factor in the use of VR relaxation. In addition, patients need to be able to personalize VR relaxation to suit their preferences and current conditions. Heyse et al [60] used a mathematical model to adapt the VR-based relaxation content to the personality profile and emotional state of the user. This could potentially create a more intuitive and less stressful starting process and use of VR relaxation. Preliminary results indicate that this approach seems usable to provide personalized emotion-based VR relaxation. Future research should focus on a more personalized version of VRelax and whether this benefits the implementation of VRelax.

To enhance the implementation of VRelax within clinical practice, several broad implementation frameworks may be used in future research. The technology acceptance model could help understand the relationship between perceived usefulness, ease of use, and the intention to use VRelax. Following the reach, effectiveness, adoption, implementation, and maintenance framework, the implementation of VRelax could benefit from investigating the adoption and maintenance of VRelax in clinical practice. The reach, effectiveness, adoption, implementation, and maintenance domains include not only the perspective of the patient but also the aspects such as how target groups are reached, how they adopt the tool, how it is implemented within specific clinical settings, and whether the intervention becomes a stable part of clinical practice [61]. The application of the normalization process theory to explore how clinicians and patients collectively integrate VRelax into care workflows, focusing on coherence (or sensemaking), cognitive participation or engagement, reflexive monitoring (experiential feedback), and collective action (shared implementation work) across organization hierarchies, could be beneficial for long-term implementation of VRelax [62].

Further research using a quantitative design, such as a randomized controlled trial, would be useful for investigating the long-term effectiveness of VRelax. Furthermore, identifying specific age and patient groups in which VRelax proves more effective by subgroup analysis could be helpful for its implementation in clinical practice. Future directions for research should also focus on the implementation of VRelax in clinical practice by investigating the perspectives of patients and health care professionals to adequately equip and train the latter. This could help determine optimal strategies to develop, implement, and promote the use of VRelax in clinical practice.

### Conclusion and Implications

This implementation study found that the themes of perceived usefulness, ease of use, and immersive factors—identified as barriers and facilitators—indicate that VR relaxation can be a valuable self-management tool for patients with psychiatric problems; however, its implementation in mental health care could be improved by focusing on personalization. This can be achieved through innovative artificial intelligence algorithms that tailor content based on personality traits or physical arousal or by improving the user interface to provide more information about the virtual natural environments and make it easier to find favorite environments. The sense of autonomy is crucial for empowering individuals with psychiatric problems, as it enhances motivation and enables self-management, making tools such as VRelax an effective complement to standard mental health care. The results highlight the importance of involving therapists in the integration of VR relaxation into mental health care by ensuring that both clients and clinicians receive adequate information and ongoing support about the therapeutic goals, safety, and suitability of VR technology for different treatment phases. Focusing on improving the usability and user-friendliness of VRelax could enhance existing facilitators. Despite the potential benefits of VR relaxation, challenges such as physical discomfort, inadequate guidance, and hardware limitations were also noted. Ultimately, maintaining the sense of autonomy, along with addressing the barriers of lack of personalization, insufficient guidance, and mixed usability, will be noteworthy in the optimal implementation of VR relaxation as a therapeutic tool in mental health care.

## Appendix

Multimedia Appendix 1 Semistructured interview guide.

Multimedia Appendix 2 Overview of the Dutch quotes.

## Abbreviations

COREQ: Consolidated Criteria for Reporting Qualitative Research
VR: virtual reality
