# Supplementary material for: Exploring the Barriers to and Facilitators of Using Virtual Reality Relaxation for Patients With Psychiatric Problems: Qualitative Focus Group Study
Source: J Med Internet Res. 2025 Jun 11;27:e65308. doi: 10.2196/65308 (PMC12198698; doi:10.2196/65308)
Supplement: Multimedia Appendix 1 [file jmir_v27i1e65308_app1.docx]

#### Appendix 1.

Table 3. Semi-structured interview guide

|  | Questions |
| --- | --- |
| Introductory question | - What is your name and what is the most relaxing holiday you have had? - If you really want to relax in everyday life, what works best for you? |
| Overarching question | - When did you get VRelax? |
| Probe question | - Think back to the first time you used VRelax, what went through your mind? - What have you experienced as positive points of VRelax per phase (startup phase, using VRelax phase, closing phase)? - What have you experienced as negative points of VRelax per phase? - How can we make the positive points even stronger? - How can we improve the negative points? - Of all the things we discussed, what do you think we should work on immediately? - Is there anything that we should have discussed that we did not? |
| Ending question | - Of all the things we discussed, what do you think we should work on immediately? - Is there anything that we should have discussed that we did not? |
