# Supplementary material for: Exploring the Barriers to and Facilitators of Using Virtual Reality Relaxation for Patients With Psychiatric Problems: Qualitative Focus Group Study
Source: J Med Internet Res. 2025 Jun 11;27:e65308. doi: 10.2196/65308 (PMC12198698; doi:10.2196/65308)
Supplement: Multimedia Appendix 2 [file jmir_v27i1e65308_app2.docx]

#### Appendix 2. Overview of the Dutch quotes

| **Number** | **Original Dutch quote** |
| --- | --- |
|  | Quotes presented in the main text of the manuscript |
| Theme 1 |  |
| 1 | “Nieuwsgierig” |
| 2 | “Verwachtingsvol” |
| 3 | “Spannend maar positief spannend” |
| 4 | “In het begin is het even zoeken. Ik ben ook gewoon benieuwd naar wat er allemaal is.” |
| 5 | “Ik raakte best wel snel verveeld.” |
| 6 | “Wat later kreeg ik ook wel een beetje dat onbestemde gevoel en ook een beetje leegheid. Al wast dingen geprobeerd en niet dat kunnen vinden wat er op dat moment heel erg aansluit.” |
| 7 | “Je komt er wel achter wat je wel en niet prettig vindt, waar je behoefte aan hebt.” |
| 8 | “Dus als ik psychotisch ben zou ik hem zeker niet opzetten, want dan zou het voor meer paniek zorgen, Vanuit de depressie die ik heb gehad, zou het wel goed werken denk ik. Als lichtpuntje of ontspanning.” |
| 9 | “Je hebt dus verschillende fasen: je hebt een pad naar depressie toe, je hebt een depressie en dan heb je er af. En ik weet in welke situatie die bril voor mij het meest optimaal is. Soms weten mensen dat ze depressief worden, dat ze het voelen aankomen, en dat ze het tegen kunnen houden als ze de bril (VRelax) gebruiken.” |
| 10 | “Ik merkte ook dat ik momenten had, dat ik VRelax had opgezet en dat het niet goed voelde voor mij. Maar als ik een stressvolle dag had gehad, dan was het weer heel prettig.” |
| 11 | “Het verschilt per keer, je hebt niet één manier. Soms moet je juist in beweging komen, soms moet je juist liggen, tot jezelf komen en soms moet je naar buiten of afleiding. Dat verschilt bij mij.” |
| 12 | “Er is één beeld, dat is een plas en daaromheen is bos. Ik ga vaak naar Terschelling en daar is ook een ijsbaan, met een plas water. Toen ik in de omgeving was, dacht ik echt van, is het daar opgenomen? Dat vond ik een hele mooie ervaring om daarvan te genieten.” |
| 13 | “Soms werd ik er gespannen van. Dat was met name bij die plaatjes waar je naar stille landschappen keek. Er kwamen dan jeugdherinneringen uit mijn moeilijke levensfase, en dat vond ik heel erg vervelend dan die op die manier naar boven kwamen door die stomme bril. Ik ben dan heel stabiel en je gaat je afvragen of je nog wel stabiel bent.” |
| Theme 2 |  |
| 14 | “Geïrriteerd, vooral in het begin omdat ik moest uitzoeken hoe het allemaal werkt.” |
| 15 | “Het activeren van de sterren en de ademhalingsoefening vroegen inspanning waardoor de ontspanning er niet was.” |
| 16 | “Onduidelijk” |
| 17 | "Onrust" |
| 18 | “Er gebeurt zoveel, maar je weet eigenlijk helemaal niet wat je allemaal tegenkomt in die filmpjes.” |
| 19 | “En misschien dat het idee erachter was: je gaat op avontuur, door de app heen, je gaat ontdekken. Maar ik heb heel vaak niet veel tijd, ik heb een gezin, ik heb werk, ik ben altijd druk. Waar ik het miste van heb op een dag is vrije tijd, dus ik wil gewoon gericht kiezen.” |
| 20 | “Ik had gehoord van dat er inderdaad ook iets van een ademhalingsoefening op staat en die wou ik zoeken. Doe er dan een kleine gebruiksaanwijzing bij want ik heb me een ongeluk gezocht. Ik was al onrustig en dan word je nog onrustiger.” |
| 21 | “Ik vond het lastig om uit het programma [VRelax] te komen. Ik bleef dan bij die twee vragen hangen. Dan kon ik wel iets uitschakelen, maar kwam ik weer bij die twee vragen terecht.” |
| 22 | “Het opstarten duurt echt heel erg lang. Ik moest dat ding op doen, aanzetten, kom in die virtuele huiskamer terecht waar ik mijn code moest invoeren. Soms gaat dat goed en soms mis. Dan moet ik nog een app uitkiezen die ik wil hebben.” |
| 23 | “Ik zou graag willen kiezen tussen één van drie minuten of één van tien minuten. Want nu vraag ik me af, hoe lang duurt dit? Past dat even in de pauze of moet ik uitgebreid gaan zitten.” |
| 24 | “Ik vind het heel positief dat het allemaal heel eenvoudig is. Ik denk dat het voor digibeten snel te leren is. […] Als je het eenmaal doorhebt, wat heel snel gaat, dan werkt het erg prettig. [...] Het nodigt hierdoor ook uit om het te gebruiken.” |
| 25 | “De ontdekking van het aanbod en hoe je erin komt, is allemaal helder en uitnodigend. Het gebruiksgemak vond ik ook ideaal.” |
| Theme 3 |  |
| 26 | “Want ik ben er zelf wel voor dat mensen zelf de ruimte hebben om te kiezen waar ze heen willen. Dus niet dat de behandelaar zegt: Nou, jij bent nu depressief, dus dan geef ik je alleen de depressiefilmpjes bij wijze van spreken. Maar dat wij als gebruikers dat zelf kunnen uitmaken werkt voor mij het beste.” |
| 27 | “Ik dacht echt van: wauw, ik kan echt even een eigen plekje vinden.” |
| 28 | “Ik heb twee kleine kinderen, de ene is best al wel groot, maar ik bedoel overdag zijn ze heel druk en dan pak je het niet makkelijk. Je hoort nog van alles om je heen. Dus die koptelefoon sluit je aan op VRelax, maar je hoort nog alles. (...) Ik deed het als de kinderen in bed lagen en voor het slapen gaan. Kon ik even ontspannen en dan ontspannen gaan slapen.” |
| 29 | “Het is puur leren ervaren wat het is en het toestel ligt ook steeds naast me, dus op het moment dat ik het wil doen kan het ook.” |
| Theme 4 |  |
| 30 | “Monotoon geluid” |
| 31 | “Irritatie” |
| 32 | “Van die olifanten, daarvan weet ik dat je daar niet zo dichtbij moet komen. Ik ben niet bang voor dieren, dat is het niet, maar ik vond het te groots. Te dichtbij. In die zin vind ik de dieren te groot.” |
| 33 | “De paarden en het contact hadden een positief effect op mij.” |
| 34 | “Ik mis dan de geuren en dacht toen van, ik heb dat eigenlijk dagelijks wel. Zoals vandaag, dan ga ik naar het park met de hond en dan zie ik daar het gras en die bloemen tijdens het struinen en dan denk ik: ja daar word ik heel ontspannen van.” |
| 35 | “Ik ergerde me dan ook aan de beeldkwaliteit waardoor ik niet in de immersie kwam.” |
| 36 | “Mijn bril is te hoog ingesteld, ook in VRelax. Dat maakt het onprettig. Het onrealistische beeld zorgt ervoor dat ik minder kan ontspannen.” |
| 37 | “Die ging gewoon door waardoor ik enorm afgeleid was. [...] Dat je in de wei staat en paarden op je af komen lopen, ik weet uit eigen ervaring dat dit erg kalmerend werkt. [...] Het lukte [VRelax] niet om dat gevoel daarbij op te wekken.” |
| Theme 5 |  |
| 38 | “Heel helder” |
| 39 | “Dus ik had echt de tijd nodig om eraan te wennen en dat heb ik ook al eerder gezegd. Dat is wel belangrijk dat mensen daarvoor gewaarschuwd worden, want ik was daar dus één persoon van die daar echt aan moest wennen.” |
| 40 | “Ik verwacht dat ik er regelmatig met een behandelaar over kan spreken, dat het een soort van vervolg heeft. We hebben dan een soort einddoel, van daar werken we naar toe.” |
| Theme 6 |  |
| 41 | “Ja ik had soms dat ik weer heel erg moest aanpassen aan waar ik was en het licht. Soms was het heel abrupt om weer in de werkelijkheid te zijn. Ik had soms het gevoeld dat ik nog niet helemaal terug was uit de virtuele wereld en daar zat dat onbestemde gevoel ook wel eens in. Ik moest echt wel vijf minuten bijkomen. Ik merkte na een paar dagen dat [...]. Als ik mijn ogen dicht deed, had ik nog een 3D ruimte om mij heen. [...] Dit is iets waar ik wel tegenop zag als ik hem weer opzette: wat zal er dit keer in mijn hoofd afspelen.” |
| Theme 7 |  |
| 42 | “De eerste paar malen was ik erg bezig met het afstellen van de bril. De druk die ik ervaar op het gezicht hield me bezig, bleef eigenlijk constant met de bril in gedachten bezig in plaats van dat de bril me daadwerkelijk hielp met eventuele ontspanningen. [...] een prominente plaats in neemt. Eigenlijk is dat jammer, het zou mooi zijn dat je de bril vergeten zou en dat je je puur in de VR wereld wanen.” |
| 43 | “Ik heb ook een lui oog en dan heb je af en toe moeite met scherpstellen en dat gaat met die bril heel moeilijk en is af en toe wel vervelend, want ik heb dan het gevoel dat ik scheel kijk.” |
| 44 | “Ik merkte ook dat ik er heel erg aan moest wennen en dat dat ook een drempel was. Ik werd er erg misselijk van en ik kreeg hoofdpijn, dus kon hem [VRelax] niet langer dan vijf of tien minuten op.” |
